# The Predisposing Factor in Dental Caries

**Published:** 1903-02-15

**Authors:** Edward C. Kirk

**Affiliations:** Philadelphia, Pa.


### DENTAL REGISTER.

Vol. LVII. February 15, 1903. No. 2.

### THE PREDISPOSING FACTOR IN DENTAL CARIES.

BY EDWARD C. KIRK, D.D.S., PHILADELPHIA, PA.

Read before the Ohio State Dental Society at its Annual Meeting, Columbus, Ohio, December, 2, 3, 4, 1902.

In studying the problem of the etiology of dental caries from a historical point of view one is struck with the tenacity with which in one form or another the belief has been maintained that the causes of caries were wholly or in part internal to the tooth. The earliest records tend to show that this conception of the cause of caries was the original explanation offered of the disease. The writings of Celsus in the first century betray his belief in the theory that decay of the teeth was caused by worms acting from within outwardly, a belief which survives among the Chinese, Japanese and Hindoos at the present time and which was undoubtedly held by them from a remote period antedating the writings of Celsus.

Later, the inflammatory theory, another aspect of the theory of internal origin, held sway and even yet is not wholly relinquished by some who have noted the reactions in the way of structural changes presented by the calcified tissues of teeth when undergoing carious action.

The modern studies of the etiology of dental caries began strictly speaking with the work of Leber and Rottenstein in

1867, when they published their important contribution entitled "Recherches sur la Carie Dentaire," followed in 1881 by the paper of Underwood and Milles, presented to the International Medical Congress of London in that year and were practically completed by the work of Prof. W. D. Miller of Berlin published in 1884. This group of brilliant scientific studies placed the active cause of dental caries before the world in its true light and established the fact that so far as the exciting cause is concerned it is external to the tooth and a factor of its environment.

The last remnant of foundation in support of an internal factor in the causation of dental caries would seem to have been destroyed by the published work of J. Leon Williams, especially his paper read before the New York Odontological Society in 1897, the British Dental Association in May 1898 and again before the New York Odontological Society in 1899.

Attacking the problem from the standpoint of the physical characters of the hard structures of the tooth Dr. G. V. Black as the result of a long and painstaking research (*Dental Cosmos* Vol. 37, page 417), felt justified in expressing the following conclusions:

"Examinations of the density, of the percentage of lime salts and of the strength of the teeth that are certainly reasonably accurate and include a sufficient number upon which to base trustworthy judgement, have shown that neither density nor the percentage of lime salts nor the strength, is in any degree a factor in predisposing the teeth to caries or in hindering its inception or progress."

Whatever may have been the differences of opinion upon minor points the major conclusion of these papers as to the exciting cause of caries being a factor of the dental environment must be generally accepted as true. The total result of our knowledge developed from the splendid researches referred to is that we know to-day *how* teeth decay. The related problem of *why* teeth decay is by no means as clear, for though we have a definite knowledge of the manner in which an infected environment may cause caries of the teeth that fact alone is wholly inadequate as a means for solving the problem of prevention of dental caries.

There are certain well-known facts, the result of general clinical observation, which our present knowledge of the cause of dental caries fails to explain. First, the immunity of certain dentures from carious action and the extreme susceptibility of others to decay; second, the periodic oscillations in the conditions of susceptibility and immunity often observed in the same individual; and third, the tendency to a physiological oscillation in immunity from tooth decay noticeable in very many individuals in passing from youth to old age.

Every dentist of some years' experience whose practice includes a number of families under continuous observation has probably noticed many instances of practically entire immunity from tooth decay, and it is reasonable to believe that the proportionate number of carious immunes is greater than those which come under professional observation; as they need no dental treatment, the dentist is not offered an opportunity to examine them.

Opposed to these cases of immunity we have those of extreme susceptibility in which, do what we will, the tooth structure melts away like snow before the noon-day sun, and between these extremes are all grades and degrees of intensity of carious action.

Again it is a commonly observed phenomenon, so common in fact that it has had a strong modifying influence upon methods of dental treatment, that many youthful dentures which in early life manifest strong tendencies to decay will later develop almost complete immunity as adult life is approached. This acquired immunity may become permanent or there may be recurrences of susceptibility at irregular intervals apparently coincident with aberrations in the nutritional status of the individual who is usually classed as one not having robust health. Finally it is frequently observed that the susceptibility of childhood has been succeeded by an immunity extending through adult life to late maturity, but as old age approaches a marked recurrence of carious susceptibility follows and if the teeth are not lost by alveolar disease they frequently succumb finally to caries.

I have endeavored merely to recall to your minds facts well-known to you as it is not my purpose to consider these peculiar oscillations in the carious process in detail. What does concern us in this connection is the fact that caries is a factor of tooth environment, and that the carious tendency varies not only as between individuals but shows equally marked variations in its intensity at different periods in the same individual. One fact illustrative of the variation in susceptibility to caries of the same individual and which has undoubtedly been noted by all present, is the frequent occurence of so-called arrested decay and eburnification of the carious surface in connection with the first permanent molars. How often is it observed that these teeth have shown a tendency to rapid decay upon their morsal surfaces at the period of their eruption and continuing possibly five or six years thereafter but gradually diminishing until it finally ceases and the exposed darkly stained dentine takes on a high polish like mahogany and the tooth is otherwise so sound and functionally satisfactory that we hesitate about placing a protecting contour filling upon it. It has been customary to explain these cases of arrested caries on the teeth in question on the theory that the decay being superficial and having destroyed the morsal surface evenly, a self-cleansing surface has been left which the triturating and detersive effect of mastication has protected from the lodgement of caries-producing bacteria, hence decay has been arrested. But we know that while certain surfaces of the teeth are by their form and position relatively immune from caries, yet in an infected and caries-producing environment no surface of any tooth is absolutely so and we must seek further for an explanation of arrested caries with eburnification of the dentine in the light of what scientific research has shown us to be the conditions which produce caries of the teeth.

These facts are known to us as the result of special bacteriological research viz: First, that caries of the teeth is due in the first instance to the action of certain groups of micro-organisms that cause acid fermentations ending mainly in the production of lactic acid which is the decalcifying agent. Second, that these lactic acid bacteria thrive best and for that matter almost exclusively in a carbohydrate medium, and third, that given a dental environment presenting the factors of the bacteria, the pabulum suited to their development and proper temperature, moisture and aerobic conditions decay of the teeth is practically certain to follow. We can conceive of but two methods by which under the conditions stated the carious process could be inhibited or prevented. First by the withdrawal or elimination of one, or more of the factors essential to the carious process, or Second, by the introduction into the environment of some substance or condition which would seriously interfere with the functional activities of the bacteria concerned in the carious process. Upon one or the other of these hypotheses it would seem that we must depend for an explanation of those cases which are either naturally or by acquired characteristics immune from dental caries.

Our prophylactic treatment has thus far practically admitted these two hypotheses in the methods generally recommended and employed for combating the progress of dental caries. All the minutiae of the dental toilet, the cleansing of tooth surfaces and the mucosa of the oral cavity are operations which, to-day, are known to be efficacious just in proportion as they eliminate bacteria and food debris, two essential factors of the carious process. While the use of buccal antiseptics is a tactic recognition of the belief that some element inimical to the functional activities of caries-producing fungi may be introduced into the oral cavity and thus prevent the results which they are known to produce when permitted to develop unmolested.

Both of these practical applications are defective in one important particular, viz: their action is temporary and they are effective only in proportion to the persistence with which they are exercised while the persistence of bacterial influence, other things being equal, is a constant factor. Eventually the constant factor of bacterial influence must produce its results when combated by the necessarily intermittent one of local hygienic and antiseptic means the degree of success of the latter being relative only to its persistency. We must seek then for a means to prevent the occurence of caries and to combat its progress when the disease is manifest by some method equally as persistent as the disorder itself, a means that will so fundamentally alter the conditions essential to a caries-producing environment that the specific micro-organisms of dental decay will be unable to thr ve therein.

The conditions which in a strict sense produce susceptibility to dental caries are but little known, while those which enter into the problem of immunity are still less known.

The researches of Miller make it very clear that the decomposition of carbohydrate food through the agency of bacteria is one of the factors, and a prominent factor, in the causation of dental caries; yet we have carbohydrate food debris in mouths immune from caries and in mouths at the same time swarming with bacteria. Or again in susceptible mouths no amount of careful cleansing or the use of buccal antiseptics will insure absolute freedom from carious attack. It would therefore seem to be clear that fermenting carbohydrate food dbris is not the only source of origin of the carious process.

The periodic oscillations in susceptibility and the tendency of some as compared with the immunity of other individuals to dental caries points strongly if not inevitably to the conclusion that dental caries is a diathetic condition. That it is closely related to the processes of general bodily nutrition there can be no doubt, for upon no other grounds can we explain the variations in the rapidity and intensity of carious action in relation to variations in the health status of the individual so generally and frequently observed. We know for example that repeated pregnancies tend to induce decay in the dentures of many women and that in wasting diseases, chronic dyspepsia and various forms of malnutrition the teeth suffer. The persistence of the impression made by the ancient error that tooth decay was a process somehow internal to the tooth has doubtless led us to feel that the interference with bodily nutrition caused by diseases are expressed as some alteration in the structure of the tooth that renders it more susceptible to caries. Since however scientific research has shown that the teeth are practically passive agents in the carious process and that the causes of caries are external to the tooth, our explanation of the manner in which pregnancy and intercurrent attacks of disease may influence susceptibility to caries must be found in the way these alterations in nutrition effect not the tooth but the environment of the tooth.

I have called attention to the two only hypotheses upon which immunity from caries can, in the light of our present knowledge, be logically explained, *i. e.*, by eliminating from the tooth environment some factor essential to the carious process, or the introduction of some element inimical to the functional activities of caries-producing organisms. That both of these conditions may and probably do result from variations in the nutritional processes from time to time, we have strong grounds for believing. We know that the mixed saliva, the fluid with which the teeth are constantly bathed, varies greatly in its composition and thanks to the earnest and painstaking work of Dr. Michaels of Paris, we are also in possession of the important generalization that the composition of saliva for the several diathetic states is fairly constant so that as the nutrition of the individual varies from time to time so likewise does the composition of the saliva. Research in this direction has not as yet progressed sufficiently far to furnish an adequate explanation of the whole question of susceptibility and immunity to dental caries unless possibly we are to receive it in a communication from Prof. Miller which he has promised for publication in the *Dental Cosmos* for January. But certain results have been obtained which are at least significant that the composition of the saliva exerts a most important influence upon the causation of caries and that in connection with the nutritional changes which influence the composition of the mixed oral secretion, we shall in due time discover the true predisposing factor of dental caries. In the examination of a considerable number of specimens of saliva I have found in all cases where true caries were prevalent but especially in the case of children the presence of a dissolved carbohydrate which responded to v. Jaksch's iodine test for glycogen or erythrodextrin. The finding of glycogen in the saliva has been previously reported by Salomon (vid. Maly's Jahresbericht, vii 55), and by Michaels (Trans. iii Congres Dentaire Internat. Paris 1900).

The close similarity of the color reaction which results from the action of the iodo-potassium iodide solution upon glycogen and erythrodextrin respectively, suggests a possible error in the reported findings of glycogen in the saliva, for the reason that the diastasic action of ptyalin upon starchy food debris is known to produce among other serial products erythrodextrin the reaction of which, with the iodine test, is scarcely distinguishable from that of glycogen.

It would doubtless be possible to determine the question with sufficient accuracy by an examination of the saliva taken directly from the glands and before contact with carbohydrate food debris in the mouth cavity was possible, that I have not as yet done owing to the necessity for special arrangements for that line of work which I am not at present ready to take up.

The significance of the presence of glycogen in this connection is of prime importance for the reason that if present in the saliva it would represent a fermentable carbohydrate produced by the process of food metabolism and dialized from the blood plasma through the salivary or buccal mucous glands into the mouth cavity and therefore were of the character of the nutritional state. On the otherhand if the substance detected in the saliva by the iodine test be simply erythrodextrin then it is not an index of the nutritional state but merely evidence of the presence of a soluble carbohydrate the product of mouth digestion.

The presence of glycogen in the saliva at times is highly probable though in the absence of a more characteristic test for that compound in the presence of erythrodextrin the presence of glycogen in the saliva can not be taken as finally demonstrated. Certain physical characteristics of the saliva, its opalescence for example, suggest in some cases the presence of glycogen, a suggestion further intensified by the chemical test referred to and the strong probability that the predisposition to dental caries is correlated with and dependent upon a diathetic state makes it appear as not at all unlikely that some fermentable carbohydrate substance such as glycogen, the product of food metabolism is constantly dialyzed from the blood plasma into the mouth through the medium of the glandular apparatus of the buccal cavity in cases of marked susceptibility to dental caries.

The reaction of the saliva is of importance as a predisposing factor of dental caries. It is a well-known fact that the growth of many kinds of bacteria is profoundly influenced by the reaction of the culture medium and until evidence to the contrary is forthcoming we are warranted in assuming that variations in the reaction of the oral fluid are not without a modifying effect upon the progress of dental caries. My own tests of saliva in pronounced carious cases show a constant alkaline or neutral reaction and that where acidity of the secretion is manifested there is a tendency to inhibition of the carious process with development of erosion.

In this connection let me call attention to the fact that these observed differences in the reaction of the saliva are characteristic of the secretion as it is poured into the mouth and are not necessarily the result of fermentative changes afterward. Hence if the reaction of the saliva has any modifying effect upon its suitability as a culture medium for the development of caries-producing fungi, we have additional evidence that the predisposition to dental caries is a factor of general nutrition, a diathetic condition dependent upon the processes of metabolism of food for upon that process depends the reaction of the saliva.

Another fact which I desire to emphasize as the result of my studies of saliva is that we are probably in error when we ascribe all cavities found in the teeth to the action of caries. My reasons for making this assertion are based upon the micro-chemical examination of many salivas from the mouths of patients in which rapid dissolution of tooth structure was taking place and notwithstanding the fact that all presented cavities affecting all of the tooth surfaces, generally speaking, a marked difference in the analytical results was observed; one class showing the existence of lactic acid action and another the action of sodium acid phosphate upon the tooth structure with little or no lactic acid action.

It is evident where tooth dissolution from any cause is progressing rapidly through acid action we would naturally expect to find the salts produced by the acid solvent upon the tooth dissolved in the oral fluids and such is the case. Again thanks to the technique of microscopic and micropolariscopic analysis given to us by Michaels we are able to determine with accuracy the existence in the saliva of those salts which are the end products of acid action upon the tooth whether the acids be produced by lactic fermentation or are due to acidity of saliva as a result of faulty metabolism in the process of nutrition.

Applying this method to the study of cases which clinically are denominated cases of rapid caries, it was soon discovered that in certain individuals little or no evidence of lactates of the tooth bases was to be found in the saliva but instead thereof an excessive amount of sodium dihydrogen phosphate and the calcium salt which is produced by its action upon the tooth. These latter cases are then strictly speaking, cases of rapid erosion and are not true caries at all, although presenting clinical appearances not unlike caries resulting in cavity formation and gradual extension of margins until the whole tooth crown is nearly or quite involved.

Investigation of the food habit and mode of life of those individuals presenting the acid saliva and the form of tooth dissolution referred to revealed the fact that they were addicted to the excessive use of carbohydrate foods, using large quantities of sweets and a minimum proportion of proteids and albuminoids; they were also of sedentary or inactive temperament disinclined to physical exertion or exercise.

It seems clear therefore in view of the abnormal existence of sodium acid phosphate in the saliva in these cases that the metabolic processes in these individuals should be characterized in general by an insufficient oxidation of the carbohydrate pabulum taken into the body, leading to an overproduction of carbon dioxid as compared with that intake of oxygen. The surplus of carbon dioxid is dammed back as it were on the blood replacing oxygen in the hemoglobin with the extreme probability that the cells of the buccal mucous glands are under these abnormal conditions of nutrition stimulated to the performance of that function which is normal to renal epithelium, viz: the conversion of neutral sodium phosphates into acid sodium phosphates through the mass action of carbonic acid, the acid exudate being in the case of the buccal mucous glands thrown out into the mouth to work out its destructive effects upon the teeth.

One other aspect of the question under consideration will bring me to the conclusion of this communication, viz: the part which natural selection may play in the predisposition to dental caries.

If we expose to the atmosphere under the same conditions two vessels one containing fruit juice for example, and the other a proteid substance like blood or meat, infusion, both will in the course of t'me undergo decomposition but the end products will in each case be totally different. The first will ferment and the process of decomposition will terminate in products acid in reaction while the second will putrefy and its decomposition will terminate in products alkaline in reaction. Both were subjected to precisely the same sources of mixed infection and we must therefore conclude that in each instance only those bacteria survived which found in the two substances respectively the kind of pabulum best adapted to their vital needs.

We have in this fact, it seems to me, the illustration of a principle, viz: that the composition of the culture medium acts passively through natural selection as a powerful agency in determining the character of bacterial infection and growth.

Applying this principle to the problem of predisposition to dental caries it seems to me obvious that as the composition of the saliva is known to vary in relation to nutritional states, we are again forced to the conclusion that the predisposing factor in dental caries is diathetic and that true prophylaxis can only be achieved by a profound study of nutrition and of those modifications in the nutritional process which so alter the composition of the oral fluids as to make them unsuitable for the propagation of caries-producing fungi, in short, to comprehend those nutritional conditions which produce immunity.

I am by no means unmindful of the importance of the recent researches of bacteriologists who have concerned themselves with the study of immunity from infectious diseases in general, more especially the magnificent work of Mechnikoff and Ehrlich, and it is not at all improbable that it may be found that the saliva of carious immunes may contain defensive proteids which prevent the action of lactic acid organisms upon tooth structure in those cases, but again it is evident that if the explanation of immunity from dental caries is to be found, for example in Ehrlich's hypothesis, both susceptibility and immunity are still factors of nutrition so that from any view-point with our present knowledge we are driven back to the conclusion that the predisposing factor in dental caries is diathetic and that local treatment alone is inadequate to arrest its ravages.

DISCUSSION.

Dr. H. A. Smith:—Abnormal saliva is undoubtedly, as the essayist suggests, an important factor in producing dental caries as well as other oral diseases. As clinicians, we have done what we could to arrest the progress of dental caries, and whilst we have not totally failed, by years of experience which has been accompanied with many unaccountable failures, we have learned that filling teeth with gold foil or other material does not necessarilly prevent the recurrence of caries. We have, however, learned that the environment of the teeth has much to do with the recurrence of caries, and we now believe that it is practicable to change this environment for the purpose of preventing recurrent caries.

Immunity to caries is a curious phenomenon, and may not always be accounted for or produced at will. I have in mind a patient who has been under my care for many years. I have probably made more than forty fillings for this patient. Some teeth have been refilled many times. It is curious however that there has never been any recurrent caries in the lower incisors. Most of the upper incisors have been refilled several times. The patient is now thirty-two years old and caries does not seem to be so prevalent,in fact I have only had to make slight repairs during the last year or two. Why do some teeth in the same mouth decay while others do not? It may be that prophylactic precautions are more effectively applied to some than to others. If it is true that caries always begin beneath the so-called bacterial plaques, and if these plaques are so concealed that they are not removed by ordinary brushing of the teeth, this explanation may be sufficient. But cases have been under observation where the bacterial plaques are really present and still no caries is produced. So we are left somewhat in doubt on this point. But no more are we in doubt here than when we attribute caries to the chemical reactions of the saliva. It has long been believed that a continuously acid saliva would favor if not really bring about the destruction in some way of the hard tooth tissues. It is also believed that a continuously over alkaline saliva is no less detrimental. It seems to me that we may not undertake scientific prophylaxis until these questions are definitely determined. Preventive therapeutics is not practicable at present. If we know that the cause of caries was a vitiated saliva, we could take such steps as might be necessary to change the character of the saliva, and so exercise the function of the physician in restoring the functions of the salivary glands to a normal condition, and so avoid the necessity of operative interference.

Dr. Taft:—There is not much doubt in my mind but that predisposing causes of various kinds are important factors in causing dental caries. These causes are more numerous and complicated when we undertake to include the complications which may be introduced by the frequent fluctuations in the saliva poured into the oral cavity by the several salivary glands, each of which may have a modifying influence.

In attempting an analysis of this hypothesis we must not forget that other factors of prime importance must also be considered. Defective or abnormal tooth structure has first of all and for a long time been considered an important consideration. In fact of so much interest has it been, that attempts to improve the structural quality of the tooth has been the subject of much thought and endeavor. It has been the experience of every one who has carefully observed this matter, that good tooth structure seemingly retards, while poor structure invites decay of the teeth. This has been so much noted that teeth have been referred to as *hard* or *soft;* and when soft, extraordinary prophylactic measures have been recommended, or expedients of various kinds have been advised to prevent or retard the progress of caries, such as filling materials of supposed therapeutic tendencies.

Lack of functional activity is a most important predisposing cause of caries. In addition to the rather indefinitely established hypothesis, that there was a greater vital resistence in a tooth which was well nourished, because of regular and systemic exercise coming from hard usage, it is a well established belief in the minds of all thoughtful observers that teeth that are kept clean by constant use are less likely to be predisposed to caries. This fact many times is advantageously utilized to preserve the teeth in mouths where, because of systemic health conditions, the saliva may be anything but normal. That systemic conditions accompanied by an impaired nervous function may produce a vitiated saliva, which of itself may prepare the way at least, for the inception of caries is also very evident, and when the saliva or mucous secretions are of such a character as to favor fermentative processes in the mouth then surely the saliva becomes an important factor in spite of all other favorable surroundings, including a vigorous function and a high development of the tooth tissues. It is commonly though that maternity is a critical time for the teeth, formerly it was supposed that the teeth suffered structurally at this time, it may be however that the result is due to the general systemic conditions which modify the oral secretions to the extent that they become inimical to tooth structure. The influence of nervous disturbances on the salivary secretions ought to be more carefully studied than it has ever been, with the intent of determing its influence in this matter. Many diseases affect the secretions of the glandular organs, and the influence of these diseases in changing the character of the saliva must be clearly outlined before we can make any intelligible effort to counteract their harmful tendencies. It may be that this can best be done by working on the saliva and carefully noting the results or tendencies of the changed conditions on the teeth. It is a large but very important study and I trust it may be prosecuted to a satisfactory issue by the essayist and others engaged with him in this work.

Dr. T. W. Brophy:—When we first began to learn of the results of bacterial function in the mouth we concluded that lactic acid produced by fermentation processes was the active, if not the predisposing, cause of dental caries. We also learned that these germs grew or developed in slightly alkaline solutions. It occured to me at that time that the proper prophylactic mouth washes were not to be found among those of an alkaline reaction, but such as would produce a neutral or acid reaction of the saliva. This I concluded would prohibit fermentation and prevent the formation of the active reagent which became the predisposing element of dental caries. If the saliva as it is poured into the mouth is the active cause of undesirable fermentative processes we should know it and adopt such means as would change it in the mouth if we can not do so in the glands, in order that it may not work mischief by its tendency to initiate dental caries by whatever process.

Dr. M. H. Fletcher:—The carnivora, snakes and other animals digest bones without difficulty. Human digestion is also capable of doing so to a limited extent. At times the secretions of all or any of the glandular organs are changed from the normal. These secretions may be only slightly modified or become actually vitiated. The saliva is no exception to this rule. It is entirely reasonable to suppose that it may at some time be sufficiently changed to at least predispose the hard tissues to decay, if it does not actually produce solution of the tooth structure, as the nascent acid which is supposed to accomplish this work results from fermentative activity.

Dr. J. S. Cassidy:—It begins to look as though we were at last beginning to get a glimpse of more light on the solution of this vexing problem of the cause of dental caries. The essayist has not attempted to overturn our present notions as to the cause in general of this disease, but has added interesting data which, when properly worked out, will undoubtedly help to clear up this subject, and put us in a position to possibly bring it under prophylactic or therapeutic control. We already know very well what the fermentative processes are and also the results. And we have thought we knew something of the peculiar environments of the human mouth which made these processes work together for good in one case and evil in another. There are evidently conditions which we have not sufficiently studied and little understood which have baffled our skill. It begins to look as though a thorough understanding of the conditions of the oral secretions in the various systemic conditions, if it be practicable to obtain an acurate diagnosis in every case, will be of invaluable assistance in enabling us to prevent destructive results. I do not think local remedies of any potency can be used exclusivly to prevent caries of the teeth. It is a larger subject, and we must take into consideration the influence of systemic conditions on the local environment before we can hope to successfully overcome this disease.

Dr. E. S. Talbott:—The work done by Dr. Miller on the bacteria of the oral cavity has established certain facts indisputably, but it evidently, from what we have heard today, has not settled the question of the etiology of dental caries, at least as to the entire cause. I have no sympathy with the idea that there is no considerable variation in tooth structure. I think there is the same variety at least of difference in hard that we note in soft tissues. And if we do not take this fact into account we shall not be able to completely solve this question. In my studies I have found there is marked variations in the tooth structures of various nations of people. English people suffer more from dental caries than any other. It is a well-known fact that we can not prevent caries by filling teeth, we only restore the tooth contour. We find some individuals almost entirely immune and others immune at times. Organs which are not in active use degenerate, and we find that the teeth are no exception. Because of the kind of food modern civilization lives upon, the teeth are becoming useless and naturely are being lost by disease and atrophy. Animals which use the teeth most and those having large pulps do not have caries.

Degenerate children do not have good teeth, because the nervous system is unstable, the teeth never devolope properly and when developed they are not used properly and the environment of the mouth is such as to facilitate the progress of decay. Degeneracy of the upper as compared with the lower teeth is due to the fact that the lower teeth are better nourished than the upper.

Dr. C. M. Wright:—This is one of the most interesting papers I have ever heard. It harmonizes with my notions of what a dentist's function should be. We have for a long time confined our efforts to preventing caries as viewed from its exciting causes. We are now, it seems to me, going back to fundamental principles and looking for predisposing causes. We must now not only know how caries comes but how we shall treat or prevent it. The changes in the oral secretions are so frequent and complicated that instruments and analytical tests of the greatest delicacy and accuracy will need be used to make adequate determinations. Quantitative as well as qualitative chemical analysis, bacteriological as well as physiological researches must be made of the secretions and these studied in their proper relations to systemic disorders, before we can hope to solve definitely this vast problem.

Dr. E. C. Kirk:—In closing this discussion I want to thank the audience for patiently hearing my paper, and those who have discussed it for apprehending my point of view of this subject, and also for the able manner in which they have amplified it.

Excessive development in fermentation will destroy the process by producing an unfavorable media for bacterial growth. The phosphate of lime however preserves the equilibrium by using up the excess of acid produced. We can not stop dental caries as Dr. Brophy suggests by using an acid mouth wash. We should produce erosion in place of taking up the fermentative acids.

I do not wish to discuss the question of defective tooth structure farther than to say that no matter how defective the tooth structure may be, it will not be subjected to caries without a bacterial fermentative environment.

In reply to the suggestion that we may learn something as to the manner in which caries is produced by remembering that bones are digested by certain animals. It is not very scientific to reach conclusions by analogy. Illustrate the danger of such a proceedure by the story of the priest who thought to reprove a drunken irishman for being drunk on the street, in saying to him, now Patrick, what would you say if you were to meet me on the street in the condition that you are? Pat quickly retorted, Your Rivirence, I would'nt say a blessed word to any one.

